# Synergistic Antibacterial Properties of Silver Nanoparticles and Its Reducing Agent from Cinnamon Bark Extract

**DOI:** 10.3390/bioengineering11050517

**Published:** 2024-05-20

**Authors:** Araceli Granja Alvear, Nayely Pineda-Aguilar, Patricia Lozano, Cristóbal Lárez-Velázquez, Gottfried Suppan, Salomé Galeas, Alexis Debut, Karla Vizuete, Lola De Lima, Juan Pablo Saucedo-Vázquez, Frank Alexis, Floralba López

**Affiliations:** 1 CATS Research Group, School of Chemical Sciences Engineering, Yachay Tech University, Urcuquí 100119, Ecuador; lgranja@yachaytech.edu.ec (A.G.A.); gott.suppan@hotmail.com (G.S.); ldelima@yachaytech.edu.ec (L.D.L.); jsaucedo@yachaytech.edu.ec (J.P.S.-V.); 2 Centro de Investigación de Materiales Avanzados CIMAV-Monterrey, Monterrey 64630, Mexico; nayely.pineda@cimav.edu.mx; 3 Centro de Investigaciones en Ciencias Microbiológicas, Instituto de Ciencias, Universidad Autónoma de Puebla, Puebla 72570, Mexico; patricia.lozano@correo.buap.mx; 4 Laboratorio de Polímeros, Departamento de Química, Facultad de Ciencias, Universidad de Los Andes, Mérida 5101, Venezuela; clarez@ula.ve; 5 Laboratorio de Nuevos Materiales (LANUM), Escuela Politécnica Nacional, Quito 170143, Ecuador; salome.galeas@epn.edu.ec; 6 Centro de Nanociencia y Nanotecnología, Universidad de las Fuerzas Armadas ESPE, Sangolqui 171523, Ecuador; apdebut@espe.edu.ec (A.D.); ksvizuete@espe.edu.ec (K.V.); 7 Departamento de Ingeniería Química, Colegio de Ciencias e Ingeniería, Instituto de Energía y Materiales, Instituto de Microbiología, Universidad San Francisco de Quito (USFQ), Quito 170901, Ecuador

**Keywords:** silver nanoparticles, nanomaterials, green synthesis, antibacterial properties, cinnamaldehyde

## Abstract

Synthesis of silver nanoparticles with antibacterial properties using a one-pot green approach that harnesses the natural reducing and capping properties of cinnamon (*Cinnamomum verum*) bark extract is presented in this work. Silver nitrate was the sole chemical reagent employed in this process, acting as the precursor salt. Gas Chromatography-Mass Spectroscopy (GC-MS), High-Performance Liquid Chromatography (HPLC) analysis, and some phytochemical tests demonstrated that cinnamaldehyde is the main component in the cinnamon bark extract. The resulting bio-reduced silver nanoparticles underwent comprehensive characterization by Ultraviolet–Vis (UV-Vis) and Fourier Transform InfraRed spectrophotometry (FTIR), Dynamic Light Scattering (DLS), Transmission Electron Microscopy, and Scanning Electron Microscopy suggesting that cinnamaldehyde was chemically oxidated to produce silver nanoparticles. These cinnamon-extract-based silver nanoparticles (AgNPs-cinnamon) displayed diverse morphologies ranging from spherical to prismatic shapes, with sizes spanning between 2.94 and 65.1 nm. Subsequently, the antibacterial efficacy of these nanoparticles was investigated against *Klebsiella*, *E. Coli*, *Pseudomonas*, *Staphylococcus aureus*, and *Acinetobacter* strains. The results suggest the promising potential of silver nanoparticles obtained (AgNPs-cinnamon) as antimicrobial agents, offering a new avenue in the fight against bacterial infections.

## 1. Introduction

In recent years, significant scientific advances in nanotechnology have led to the development of many nanomaterials with customized properties for applications in various domains. These applications span biotechnology, catalysis, optics, electronics, textiles, and the food industry [1,2,3,4,5,6,7]. Silver nanoparticles have attracted considerable attention in the field of nanomaterials due to their widely documented outstanding optical, magnetic, and electrical properties, which emerge from the peculiar behavior exhibited by matter at the nanometer scale [8,9,10,11]. Specifically, the well-documented antibacterial activity associated with silver has prompted the incorporation of silver nanoparticles in various applications, with the purpose of addressing the challenges arising in the post-antibiotic era. This phenomenon has stimulated the development of new agents capable of combating pathogenic microorganisms without promoting the emergence of additional resistance mechanisms [12,13,14,15,16,17,18,19].

Unlike physical methods, which usually require specialized equipment, chemical synthesis methods offer a more accessible and less demanding alternative in terms of experimental requirements. These approaches involve meticulous formulation design that requires the selection of chemical reagents and precise control of experimental parameters to achieve control of the desired size and shape through assembly or self-assembly [20,21,22,23]. However, some of these chemical reagents are expensive and pose a risk to the environment. To address these drawbacks and take advantage of the inherent chemical structures of biological systems, green synthesis methods have gained appeal as an alternative to produce silver nanostructures [24,25,26,27,28,29,30,31,32,33,34,35,36]. Methods using plant extracts (roots, leaves, stems, fruits), microbial cells (yeasts, bacteria, fungi), or biopolymers as reducing agents have been investigated. This strategy offers the additional advantage of potentially conferring antibacterial activity to the resulting nanomaterials [37,38,39,40,41,42,43]. The higher stability and efficiency observed in bio-assisted nanostructures are attributed to the intricate chemical structures of the bioagents involved, which lead to more complex mechanisms in the reduction and limitation processes [25].

Several natural extracts have been evaluated for their potential to reduce silver ions and facilitate the formation of nanomaterials with diverse sizes and morphologies, suggesting favorable synthesis routes for obtaining silver nanoparticles [25]. Extracts derived from *Cinnamomum zeylancium*, *Acorus calamus*, Tea, Cocous and *Nelumbo nucifera*, *Pistacia atlantic*, *Premna herbacea*, *Centella asiatica*, *Acalypha indica*, *Allium sativum* leaves and *Citrus sinensis*, *Vitex negundo* [30,39,44,45,46] have been used as reducing agents, giving rise to spherical silver nanoparticles with diameters ranging from 8 to 50 nm. On the other hand, Aloe Vera extract [28], *Memecylon edule,* or *Eclipta prostrate* [47] extracts have also produced silver nanoparticles with triangular and hexagonal shapes, ranging from 25 to 80 nm in size. Furthermore, the *Datura metel* extract yielded quasi-linear superstructures with sizes between 16 and 40 nm [27,48]. However, conventional agents from natural extract only reduce the number of agents without additional bioactivity.

Medical applications of silver nanoparticles range from antimicrobial and anticancer treatments to wound healing, bone repair, vaccine adjuvants, anti-diabetic agents, and biosensors [43]. It is notable that the biological activity of silver particles is enhanced at subnanometer scales (silver Angstrom particles, AgAPs), opening the research landscape in the applications of AgNPs and sub-AgNPs [49,50]. Recently, Khan et. al. published a review of applications of AgNPs in agriculture, acting as antivirals, antibacterials, antifungals, and even as nano-pesticides. Interestingly, they improve seed germination and plant growth and also improve the quantum efficiency of the photosynthetic process [51]. In the field of material science, silver nanoparticles are recognized for their versatility in applications in biomolecule sensing, diagnostic in healthcare systems, optics, and electronics applications. A particular example of the use of AgNPs in optics is the development of metasurfaces formed of self-assembled silver nanocubes (AgNCs) immobilized on a thick layer of gold, generating a new generation of dynamically controlled optical components [52].

Many studies have demonstrated the effective bactericidal capacity of silver nanoparticles (AgNPs), which operate through several simultaneous mechanisms of action. Some promising results have shown that smaller nanoparticles exhibit greater bactericidal activity [14,48,53,54], mainly attributed to their greater surface area available for interaction with bacterial membranes. This interaction leads to membrane disruption, protein dysfunction, oxidative stress, and DNA damage within bacteria, altering essential functions such as permeability and respiration [3,55,56,57,58], ultimately resulting in bacterial death. The proposed mechanisms for DNA modification suggest the participation of silver ions, which can hinder protein synthesis, deactivate respiratory enzymes, and generate reactive oxygen species, thereby interfering with adenosine triphosphate (ATP) production. Nanostructures with smaller sizes and spherical or quasi-spherical shapes are more prone to release silver ions due to their larger surface area [8,59].

Incorporating phytochemical agents, such as organic compounds or antibiotics, in conjunction with silver nanomaterials has demonstrated a synergistic effect against pathogenic bacteria [14,60]. This innovative approach of combining phytochemicals and metallic nanomaterials has emerged as a promising strategy to address the challenge of multi-drug resistance technology (MDR) and meet the pressing need for effective antibacterial agents [61,62]. Using nanostructures improves the bioavailability of phytochemical agents, facilitating their controlled release at the desired target site or tissue and improving the stability, antimicrobial efficacy, and reduced toxicity to the host [63,64,65]. Cinnamaldehyde, the main chemical component extracted from cinnamon bark and its essential oil, belonging to the Cinnamomum genus of the Lauraceae family, has a wide range of beneficial properties, including analgesic, antiseptic, insecticidal, and parasiticidal effects. Due to its exceptional antimicrobial efficacy against various infections [61,66], cinnamaldehyde can be considered a promising phytochemical agent. When used as a reducing and capping agent during the synthesis of silver nanostructures, a redox reaction, as depicted in Equation (1), is expected to occur, converting cinnamaldehyde into cinnamic acid. By synergistically combining the phytochemical activity of cinnamaldehyde with the antimicrobial properties of silver nanoparticles, the combined system exhibits enhanced antibacterial efficacy [67,68,69].

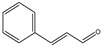
 + 2 Ag+ +2 OH−- → 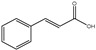 + 2 Ag0 + 2 H2O(1)

In this work, a one-pot method of green synthesis was employed to produce silver nanoparticles, only involving two reagents, namely a silver nitrate solution and an emulsified cinnamon bark extract. The extract derived from cinnamon bark served as a sustainable and natural source of phytochemical compounds, acting as reducing and capping agents during the formation of the silver nanoparticles. The morphological, structural, and chemical features of the resulting silver nanostructures were evaluated, as well as their bactericidal activity against different resistant and sensitive Gram-negative and Gram-positive bacteria. This simple synthesis method represents a promising alternative for generating nanocomposites with significant applications in the food and biotechnology industry.

## 2. Materials and Methods

Silver nitrate (AgNO_3_) was purchased from Sigma Aldrich (99.5%) and used as obtained without further purification. The cinnamon barks were purchased from local stores, and its extract was acquired by steam distillation for 2 h, maintaining a 1:9 (*w*:*v*) ratio of crushed cinnamon bark mass and distilled water volume. The cinnamaldehyde was extracted by (steam) distillation and then stored for later use and analysis.

Bacterial strains used: *Klebsiella pneumoniae* (KpCL17), *Escherichia coli* (DH5α), *Pseudomonas aeruginosa* (PAO1), *Acinetobacter haemolyticus* (AN54), *Klebsiella pneumoniae* (KpE52), *Escherichia coli* (C7230), *Pseudomonas aeruginosa* (PE52), *Acinetobacter* (AN2) and *Staphylococcus aureus* (29213), came from the Pediatric Hospital of the city of Puebla in Mexico.

### 2.1. Preparation of Silver Nanoparticles from Cinnamon Bark Extract (AgNPs-Cinnamon)

Silver nanostructures were prepared by mixing the cinnamaldehyde extracted with a 1 mM AgNO_3_ solution, maintaining a 1:1 (*v*:*v*) ratio. The mixture was stirred for 20 min at 40 °C. The onset of the reaction was evidenced by a light yellow/pink color After preparation, the resulting colloidal dispersion was carefully stored for later use.

### 2.2. Characterization of Silver Nanoparticles (AgNPs-Cinnamon)

FTIR spectroscopy analysis was carried out to identify specific functional groups of cinnamaldehyde from the extract and the resultant AgNPs-cinnamon, from which it is possible to assess the structure chemical alterations experienced by cinnamaldehyde after the oxidation process during silver reduction. FTIR spectra were obtained using a Perkin Elmer/100 FTIR spectrophotometer (San Miguel de Urcuquí, Ecuador), operating in a range between 500 and 4500 cm^−1^.

GC-MC analysis to confirm the presence of cinnamaldehyde was performed with a SYNAPT-G2Si Waters mass spectrometer (San Miguel de Urcuquí, Ecuador) coupled to an Agilent 7290A Gas Chromatography (San Miguel de Urcuquí, Ecuador) equipped with an Agilent DB5-MS (San Miguel de Urcuquí, Ecuador), 30 m length, 0.25 mm I.D., 0.25 µm (5% phenyl and 95% polydimethylsiloxane) column. The carrier gas was Helium (1.5 mL/min), and a temperature gradient of 70 °C to 300 °C (5 °C/min) was used for the analysis. Furthermore, the analysis of cinnamon bark extract involved various phytochemical tests aimed at identifying the presence of proteins, phenols, and flavonoids. The detailed methodologies for these tests and the spectroscopic characterization of cinnamaldehyde can be found in the Supplementary Materials (S1).

The formation of nanoparticles was tracked through observation of the Surface Plasmon Resonance (SPR) phenomenon by UV Vis spectroscopy. This phenomenon generates signals in the visible region that can be recorded in the UV-Vis spectrum. A Perkin Elmer/LAMBDA 1050 UV/Vis spectrophotometer (175 to 3300 nm, San Miguel de Urcuquí, Ecuador) with a quartz cuvette was used for recording spectra and evaluating wavelengths between 300 and 800 nm with a scan speed of 5 nm/s and a resolution of 1 nm.

The shape and structure of the AgNPs-cinnamon were determined by Transmission Electron Microscopy (TEM) using a microscope model Tecnai G2 Spirit Twin (Sangolquí, Ecuador) equipped with an Eagle 4k HR camera (Sangolquí, Ecuador). Briefly, the solid samples were resuspended in absolute ethanol. 5 µL of each suspension was placed on a Copper F/C TEM grid. Images were acquired at different magnifications by operating the microscope at 80 kV. Additional morphology evaluation was performed using a TESCAN model MIRA 3 field emission scanning electron microscope FEG-SEM. Images were obtained at various magnifications operating at 10 kV. Each of the samples was placed on a scanning electron microscopy pin fixed with a double layer of double-sided carbon tape.

Dynamic Light Scattering (DLS) was performed on nanoparticles suspended in a liquid phase to determine their size distribution. For this analysis, MALVERN, Zetasizer ZS90 automatized equipment (Coatzacoalcos, Veracruz, México) with DTS0012 cells (Malvern Panalytical, Coatzacoalcos, Veracruz, México) was used. ζ-potential of the obtained nanostructures was additionally evaluated on the same equipment. From these measurements, it is possible to determine the stability of the prepared nanoparticle system.

### 2.3. Bactericidal Activity Analysis

The AgNPs-cinnamon bactericidal activity was evaluated for Gram-negative and Gram-positive bacteria of resistant and sensitive types using both disk diffusion methods, commonly referred to as the Kirby Bauer method, and the microdilution in broth method. The disk diffusion method was performed by inoculating a standard quantity of microorganisms on a plate with the Muller Hinton Broth (MHB) base, forming bacterial turf. This method implies the determination of the generated zone of inhibition (ZI), in which the effectiveness of antibiotics or bactericides against specific bacterial strains is estimated from measurements of that inhibition zone [42]. ZI is the area around an antibiotic- or bactericide-impregnated disk where bacterial growth is visibly inhibited. Therefore, this method provides a qualitative assessment of the bacterial susceptibility of the tested substance. The disk diffusion method is widely used and provides a quick way to determine the effectiveness of various antibiotics and bactericides against different bacterial strains. The resistant bacterial cultures *Pseudomonas aeruginosa* (PE52), *Acinetobacter haemolyticus* (AN54), *Escherichia coli* (C7230), and *Staphylococcus aureus* (29213), which came from the Pediatric Hospital of the city of Puebla in Mexico, were used to evaluate the antibacterial properties of AgNPs-cinnamon.

Under appropriate conditions, sensi-disks were prepared by impregnating 1 cm diameter filter paper discs with 10 µL of various antibacterial agent samples, including silver nitrate solution and cinnamon extract, denoted below as Blank 1 and Blank 2, respectively, and AgNPs-cinnamon suspensions concentrated 2, 3, 5 and 10 times their initial concentration. These impregnated discs were placed in Petri dishes containing inoculated bacteria and incubated at 37 °C. In all the cases, a disk containing 10 mg of ampicillin was included as a growth inhibitor reference. Three replicates were conducted for each sample.

Mueller Hinton Broth (MHB) method was followed as previously described [70]. Briefly, in a 38 g/L MHB culture medium, the bacteria were inoculated. The Sensi-disks impregnated with the bactericidal agent samples were placed on the inoculated culture medium, and the agar was gently pressed to ensure that they were sufficiently impregnated on the surface. After an incubation process at 37 °C for 18 to 24 h, the disks were removed, and the inhibition halos were measured.

The broth microdilution method involves creating a liquid broth with varying antimicrobial agent concentrations. Microorganisms were introduced, and after incubation, growth was monitored. During this method, the bacterial strains were standardized to 0.5 McFarland [70]. 100 µL of Luria-Bertani (LB) culture medium was poured into the appropriately labeled wells, adding 100 µL of respective AgNPs-cinnamon samples and 10 µL of bacteria inoculum. The incubation process at 37 °C took 20 h. Gram-negative sensitive bacteria *Klebsiella pneumoniae* (KpCL17), *Escherichia coli* (DH5α), *Pseudomonas aeruginosa* (PAO1), and *Acinetobacter haemolyticus* (AN54), as well as the Gram-negative resistant bacteria *Klebsiella pneumoniae* (KpE52), *Escherichia coli* (C7230), *Pseudomonas aeruginosa* (PE52) and *Acinetobacter* (AN2) were used to evaluate the antibacterial capacities of the AgNPs-cinnamon by this method.

## 3. Results and Discussion

### 3.1. Phytochemical Tests of Cinnamon Extract Cinnamon

The phytochemical tests presented in the Supplementary Materials (Figure S1) indicate that the extract obtained from the steam distillation of cinnamon bark mainly comprises cinnamaldehyde with a small quantity of flavonoids and alkaloids. Unlike other previously reported works such as that of Ahmad et al. in which the reducing and capping agents of silver ions are attributed to the presence of phenols [39]. The silver nanoparticles system was prepared from hydro-distilled steam distillation, containing mainly cinnamaldehyde.

### 3.2. FTIR and GC-MS Characterization of Cinnamon Extract and AgNPs-Cinnamon

**Figure 1:**
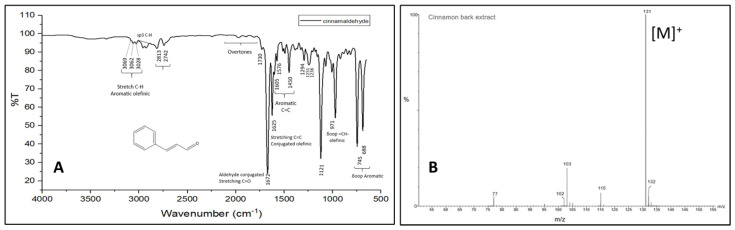
(**A**) FTIR spectrum and (**B**) GCMS spectrum of cinnamaldehyde extracted from cinnamon bark.

Following the separation of the oily phase from the semi-transparent white microemulsion obtained from the steam distillation of cinnamon bark, FTIR spectroscopy analysis was directly carried out to the obtained distillate, as well as to the purified and decanted aqueous phase. The resulting FTIR spectrum for purified cinnamaldehyde is presented in Figure 1A. The most prominent peaks in the spectrum can be attributed to the distinctive features of cinnamaldehyde. At 3028, 3062, and 3069 cm^−1^ due to the aromatic and olefinic stretching vibration of C-H groups. Additionally, at 2813 and 2742 cm^−1^, the Fermi resonance of the C-H of the aldehyde is observed. The peak at 1673 cm^−1^ corresponds to the stretching of the conjugated carbonyl group C=O of the aldehyde, while the peak at 1625 cm^−1^ is associated with the stretching C=C of the conjugated olefin. The peak at 973 cm^−1^ is characteristic of C-H out-of-plane (oop) bending of a disubstituted trans-olefin group. The peaks between 1605 and 1451 cm^−1^ are associated with the vibrations of C=C in the aromatic structure. Furthermore, the peaks at lower wavenumbers at 744 and 688 cm^−1^ indicate a monosubstituted benzene’s aromatic structure. Finally, a peak at 1121 cm^−1^ due to the stretching of the alpha carbon–aldehydic carbon C-C bond is also indicated. In addition, HPLC and GC-MS of the microemulsion were developed to evaluate the composition of the extract; both chromatograms are presented in the Supplementary Materials (Figures S2 and S3). The mass spectrum taken from the peak at 22.3 min of retention time of the GC-MC chromatogram is shown in Figure 1B, demonstrating the high purity of cinnamaldehyde by the presence of their characteristic fragmentation pattern (*m*/*z* = 131, 103, 77, and 51) with the molecular ion and base peak at *m*/*z* = 131 as has been reported before [45].

**Figure 2:**
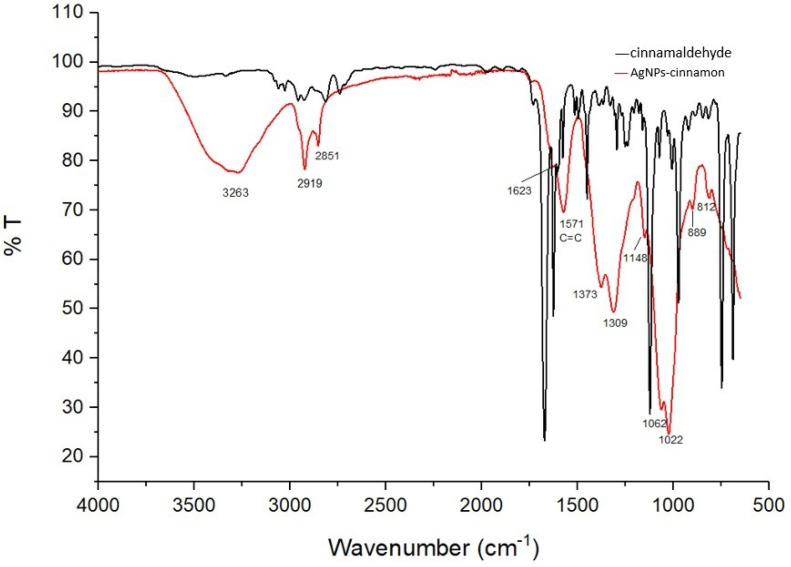
FTIR spectra of cinnamaldehyde extracted from cinnamon bark (black line) and the colloidal dispersion of synthesized silver nanoparticles, AgNPs-cinnamon (red line).

Figure 2 shows a comparison between the FTIR spectra of the extracted cinnamaldehyde and the solution resulting from the preparation of silver nanoparticles to explain the oxidation of cinnamaldehyde to cinnamic acid. Some differences in the spectra can be observed, i.e., the appearance of the peak at 3263 cm^−1^, which could evidence the presence of O-H associated with the carboxylic acid, the disappearance or attenuation of the peaks at 2813 and 2742 cm^−1^ related to the C-H of aldehydes, as well as the appearance of the peak at 1148 cm^−1^ corresponding to stretching of C-O. These differences are clear evidence of the presence of carboxylic acid and that the cinnamaldehyde acts as a reducing agent in the formation of AgNPs-cinnamon. Additionally, it is important to highlight the presence of the double peaks related to the symmetrical and asymmetrical tensions of the carboxylates occurring at 1623–1571, 1373–1309, and 1062, and 1022 cm^−1^, which explains their interaction with silver nanoparticles. The bimodal shape of these peaks suggests different size and shape distributions of silver nanoparticles. These results agree with what was previously reported by Premkumar et al. [71]. However, for the low energy bands, Premkumar’s work reported several peaks in the region of 520 cm^−1^ that were assigned to vibrations of carbon halides (C-Cl, C-Br, C-I). In contrast, we observed a single broad peak at 695.87 cm^−1^ associated with the C-H aromatic bending as mentioned above.

### 3.3. Surface Plasmon Resonance Analysis by UV-Vis Spectroscopy

**Figure 3:**
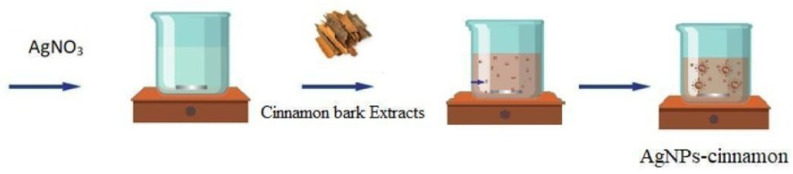
Scheme of synthesis of silver nanoparticle from cinnamon bark extract.

**Figure 4:**
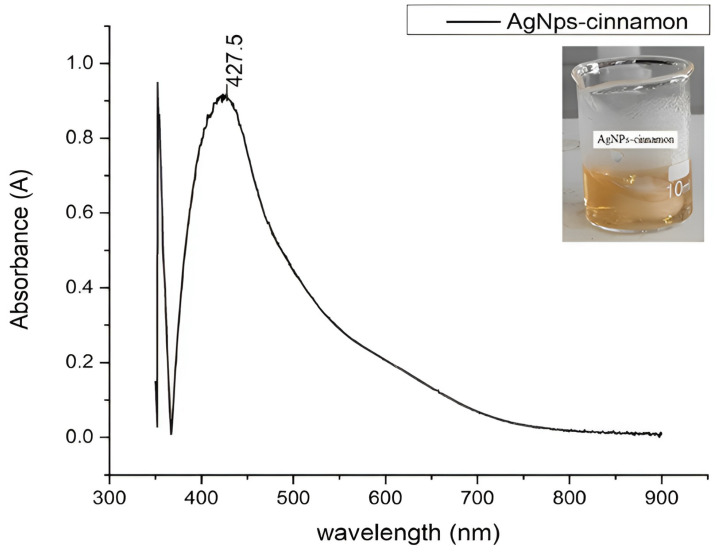
UV-Vis spectrum of the product of the green synthesis of AgNPs-cinnamon.

The formation of the silver nanoparticles could be evidenced by the change in coloration presented by the colloidal dispersion obtained, as represented in Figure 3. This was further confirmed by the appearance of strong absorption bands 427.5 nm in the UV-Vis spectrum, as is shown in Figure 4, due to the collective oscillations of the conduction electrons of metal nanoparticles that come into resonance with the electromagnetic radiation with which the AgNPs are excited. This phenomenon of Surface Plasmon Resonance (SPR) can be considered as the signature optical property of noble metal nanoparticles [22,23,65]. The UV-Vis spectroscopy analysis allows not only the confirmation of the presence of silver nanoparticles but also the estimation of their shape [8,71]. In some cases, when describing nanoparticles, the term Localized Surface Plasmon Resonance refers to a particular kind of SPR in which the electromagnetic field remains localized in a nanoscale pattern surface [72]. The redshift from 400 nm implies the presence of shapes other than spherical nanoparticles due to surface faceting [5,71,72,73].

In addition, a subtle shoulder can be distinguished at higher wavelengths, suggesting the existence of bigger silver nanoparticles with different shapes, showing the ability of the cinnamaldehyde extracted as a reducing and capping agent [8].

### 3.4. Morphological Analysis

**Figure 5:**
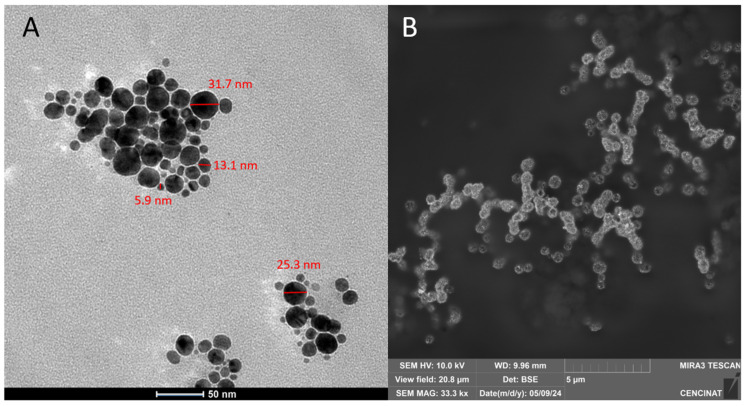
(**A**) TEM and (**B**) SEM images of AgNPs prepared from the cinnamon bark extract.

TEM and SEM micrograph images of the synthesized AgNPs are displayed in Figure 5A and 5B, respectively. According to the shown image, the particle sizes of AgNPs ranged from approximately 5.9 to 31.7 nm. Figure 5A displays TEM micrographs illustrating the presence of AgNPs-cinnamon of different shapes, mostly spherical particles, of varying sizes. SEM image in Figure 5B shows structures beyond the spherical shape, confirming the red shift of the surface plasmon resonance (427.5 nm). The formation of silver nanoparticles confirms the reducing activity of cinnamaldehyde, the primary constituent of cinnamon bark extract, as discussed earlier.

The shape of silver nanoparticles has been recognized as a critical factor influencing their bactericidal activity [74,75,76]. This phenomenon can be attributed to the shape-dependent disruption of cell membranes in Gram-negative bacteria, such as *E. coli* [77,78]. Alshareef et al. [79] have reported that silver nanoparticles in both spherical and rod shapes exhibit strong antimicrobial properties. However, Cheon et al. [80] observed variations in the bactericidal effectiveness of silver nanoparticles following this sequence: spherical > disc > triangular nanoparticles. In this regard, the presence of a combination of different shapes of AgNPs-cinnamon, as seen in Figure 5, could offer advantages in terms of bactericidal activity. This is because different shapes of nanoparticles may interact with bacterial cells in diverse ways, potentially leading to multiple mechanisms of cell disruption.

**Figure 6:**
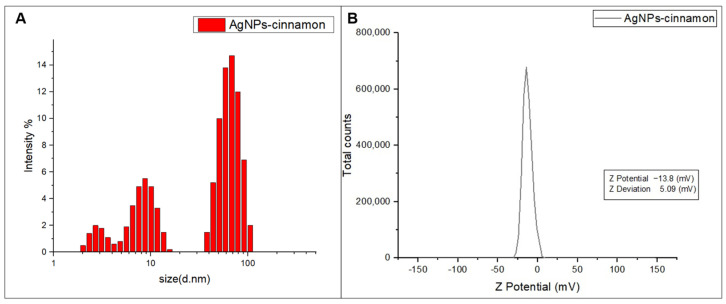
(**A**) Size of AgNPs-cinnamon measured by Dynamic Light Scattering, and (**B**) ζ−Potential determined for AgNPs-cinnamon.

DLS analysis provides a graphical representation of the different sizes of the AgNPs-cinnamon obtained, along with their corresponding intensities. This illustration is shown in Figure 6A, in which three peaks can be distinguished, each with a different percentage of intensity. Each of these peaks, occurring at 2.94 nm, 8.7 nm, and 65.1 nm, are related to variable dimensions of silver nanoparticles in the sample. The effective diameter corresponds to 44.8 nm for a distribution polydispersity of 0.356. These results reveal a polydispersity in the nanoparticles, as observed by electron microscopy analysis and suggested by the FTIR spectroscopic analysis, supporting the idea of the presence of nanoparticles of different shapes.

By analyzing the ζ−potential, it is possible to examine the potential associated with the surface of nanoparticles, as well as to attest to the stability of the nanoparticles, as long as the ζ−potential values range between −30 mV and 30 mV [81]. The results obtained from this analysis are shown in Figure 6B, from which it is feasible to see that the AgNPs-cinnamon has a negative surface charge of −13.6 mV. This surface electrical potential value indicates the stability of AgNPs-cinnamon, as evidenced by the observed absence of noticeable changes in these systems after several months.

### 3.5. Evaluation of Antibacterial Properties

**Figure 7:**
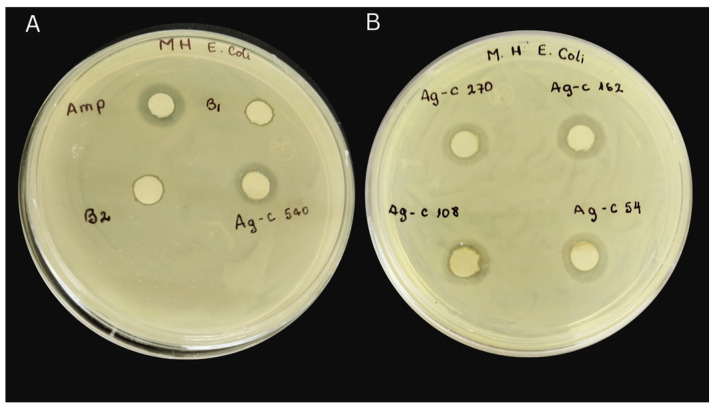
Disk diffusion assay results evaluating the effect of concentration of AgNPs on bacterial growth after incubation at 37 °C for 24 h on agar plates. (**A**) Amp = 10 µg ampicilin, B1 = 1.7 µg AgNO_3_, B2 = 20 µL cinnamon bark extract, AgC-540 = AgNPs-cinnamon 540 µg/mL. (**B**) AgC-270 = AgNPs-cinnamon 270 µg/mL, AgC-162 = AgNPs-cinnamon 162 µg/mL, AgC-108 = AgNPs-cinnamon 108 µg/mL, AgC-54 = AgNPs-cinnamon 54 µg/mL.

Results of the study of the bactericidal activity of the AgNPs-cinnamon and their precursors are shown in Figure 7 and in Table 1, the diameters of the inhibition zones are indicated. The control samples; silver nitrate (Blank 1) and cinnamon extract (Blank 2), show small zones of inhibition, suggesting a slight bactericidal capacity against Gram-negative and Gram-positive bacteria of silver ions and cinnamon extract.

Inhibition halos are present even at the lowest concentrations of AgNPs-cinnamon (54 µg/mL). As the concentration of the nanoparticles increases two and three times from its initial concentration, halos of greater diameter are observed (Table 1), indicating an increase in their bactericidal capacity (see Figure 7). This effect is further accentuated at higher concentrations of AgNPs showing inhibition halos that reach notable sizes, for example, the system AgNPs-cinnamon 540 µg/mL cause inhibition zones up to 15 mm in diameter for PE52, 14 mm for AN54, 15 mm for C7230, and 10 mm for 29213 bacteria. It is interesting to note that by increasing the AgNPs-cinnamon concentration from 270 µg/mL to 540 µg/mL, the values of the inhibition halos remain almost constant, suggesting that the maximum antibacterial capacity of the evaluated system is reached. In summary, these results reveal that silver nanoparticles synthesized from cinnamon extract have bactericidal properties that vary with the AgNPs concentration, reaching their maximum efficacy at moderate concentrations, beyond which no substantial increase in bacterial inhibition is observed.

**Figure 8:**
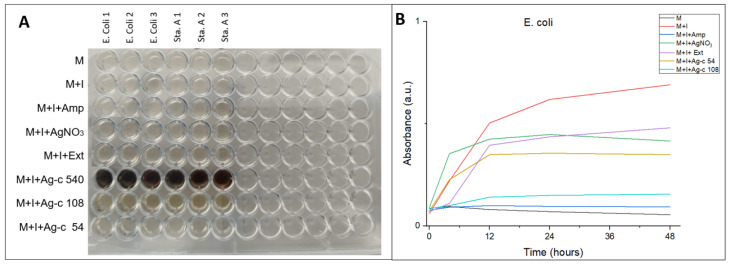
ELISA microplates of microdilution in broth assays (**A**) of AgNPs-cinnamon against *S. aureus* as a function of time (**B**). Different AgNPs concentration: 54 µg/mL and 108 µg/mL were evaluated. (M = medium, I = Inoculum, Amp = Ampicillin, Ext = Cinnamon bark extract).

The result of the broth microdilution method is shown in Figure 8A for analysis of the antibacterial properties of AgNPs-cinnamon. By introducing the different sensitive and resistant bacteria tested into the wells, bacterial growth is clearly observed (Figure S5 in Supplementary Materials). In addition, the appearance of turbidity is distinguished, which indicates the proliferation of colonies. A similar phenomenon of bacterial growth is evident in the case of cinnamon extract, except for PE52 Pseudomonas bacteria, whose growth is inhibited by the cinnamon extract. However, as is detailed in Table 2, a marked antibacterial activity is appreciated for all of the tested bacteria in the wells with AgNPs-cinnamon since no bacterial growth is observed in any way. Li et al. examined the activity of AgNPs against *P. aeruginosa*, *S. epidermidis*, and *E. coli*, demonstrating that AgNPs are more active against *E. coli* than *P. aeruginosa* and *S. epidermidis* [82]. However, it is important to consider the dependence of antibacterial activity on the concentration of silver nanoparticles. From the various studies on MDR bacteria, AgNPs are effective against pathogenic bacteria such as *E. coli*, *S. Typhi*, *S. epidermidis*, and *S. aureus*, *P. aeruginosa* [70,82,83,84,85].

To evaluate the dependence of concentration on antibacterial activity, broth microdilution. Three different concentrations of silver nanoparticles were tested: Ag-c 54, Ag-c 108 and Ag-cc 540. The antibacterial activity was evaluated by measuring absorbance at a wavelength of 630 nm during 48 h, whose results are shown in Figure 8B. It is observed that silver nanoparticles with higher concentrations significantly inhibit bacterial growth.

The effect of AgNPs-cinnamon on the growth kinetics of *S. aureus* is shown in Figure 8B. Different growth stages can be identified, while the decline stage cannot be attained because the living and dead bacterial cells show absorbance at 630 nm [86]. The silver nitrate (Blank 1) acts as a bacteriostatic agent, attaining a constant stationary phase of bacteria growth at 12 h, while the cinnamon bark extract (Blank 2) does not reach it. On the other hand, the presence of AgNPs-cinnamon notably affects the growth of bacteria. The sample Ag-c 54, corresponding to 54 µg/mL AgNPs, exhibits a decrease in the absorbance, implying a decrease in the number of live bacterial cells. Furthermore, a higher concentration of AgNPs (108 µg/mL) results in a higher bacteriostatic behavior.

## 4. Conclusions

In this work, the synthesis of silver nanoparticles with antibacterial activity using cinnamon bark extract as a reducing and capping agent was successfully achieved. The process involved mixing the cinnamon extract, mainly composed of cinnamaldehyde, with a silver nitrate solution in a specific ratio, leading to the generation of silver nanoparticles (AgNPs-cinnamon). The formation process of AgNPs-cinnamon is mainly attributed to the interaction between silver nitrate and cinnamaldehyde and not with other constituents present in the cinnamon extract. This conclusion arises from phytochemical and spectroscopic analyses of the cinnamon extract, which consistently yielded negative results for the presence of protein and phenol compounds. These results are consistent with the GC-MS and HPLC analysis that confirm the high purity of cinnamaldehyde. However, the presence of a small content of flavonoids and alkaloids was confirmed, providing the appropriate conditions for the reduction of silver and the subsequent formation of silver nanoparticles. FTIR analysis revealed chemical modifications that occur in the cinnamaldehyde during the silver reduction process, with the appearance of carboxylic acid groups, supporting the proposed oxidation of cinnamaldehyde to cinnamic acid and their interaction with the silver nanoparticles. In addition, UV-Vis spectroscopy corroborated the presence of silver nanoparticles by detecting surface plasmon resonance (SPR) signals within the visible spectrum. The spectrum also suggests the coexistence of various shapes and sizes of nanoparticles.

The shape and size of AgNPs-cinnamon, analyzed by DLS, TEM, and SEM imaging, confirmed their morphological diversity. This variety of forms could potentially exert an influence on its bactericidal activity. Meanwhile, the DLS measurements indicated a polydispersity in the size of the nanoparticles, with several peaks corresponding to different dimensions. ζ-potential analysis indicates that AgNPs-cinnamon possesses a high enough surface charge, implying good stability.

An evaluation of the antibacterial properties of AgNPs-cinnamon was carried out through two different methodologies: disk diffusion and broth microdilution methods. It is important to mention that AgNPs-cinnamon showed bactericidal activity against various Gram-negative and Gram-positive, drug-sensitive, and drug-resistant bacteria. The bactericidal efficacy was directly correlated with the concentrations of AgNP-cinnamon. By contrast, cinnamon extract alone had minimal impact on bacterial growth. The morphological diversity exhibited by AgNPs-cinnamon is potentially important for improving its efficacy against different bacterial strains.

## Figures and Tables

**Table 1:** Results for antibacterial activity tests of AgNPs-cinnamon dispersions at different concentrations, silver nitrate (Blank 1), and cinnamon extract (Blank 2).

	Zone of Inhibition (mm)			
Sample	C7230	PE52	AN54	29213
Blank 1: AgNO_3_	5.2	5.1	5.1	5.2
Blank 2: Cinnamon bark extract	6	6	6	6
AgNPs-cinnamon 54 µg/mL	7	9	6	6
AgNPs-cinnamon 108 µg/mL	15	10	7	6
AgNPs-cinnamon 162 µg/mL	13	10	7	7
AgNPs-cinnamon 270 µg/mL	16	14	14	10
AgNPs-cinnamon 540 µg/mL	15	15	14	10

**Table 2:** Antibacterial activity of cinnamon bark extract and AgNPs-cinnamon dispersions. Symbols ‘−’ and ‘+’ denote no antibacterial activity and high antibacterial activity, respectively.

Sample	KpE52	KpLC1	C7230	DH5α	PAO1	PE52	AN2	AN54	29213
Medium and Inoculum	−	−	−	−	−	−	−	−	−
Cinnamon bark extract	−	−	−	−		+	−	−	−
AgNPs-cinnamon	+	+	−	+	+	+	+	+	+

## Data Availability

The data presented in this study are available on request from the corresponding author.

## Supplementary Materials

The following supporting information can be downloaded at: https://www.mdpi.com/article/10.3390/bioengineering11050517/s1, Figure S1. UV-Vis spectrum of cinnamon bark extract. Figure S2. Chromatogram of cinnamon bark extract on the HILIC column. Peak 1 represents cinnamaldehyde and peaks 2 and 3 represent two minor components of the extract. Figure S3. GC chromatogram showing the characteristic base peak of cinnamaldehyde m/z = 131 at a retention time of 22.3 min. Table S1. Results of the determination of secondary metabolites in the cinnamon bark. Figure S4 Phytochemical screening of qualitative tests to secondary metabolites. Figure S5 ELISA microplate of broth microdilution method to analyze the antimicrobial activity of silver nanoparticles for the tested bacteria. Figure S6 Bacterial viability at 54, 108, and 540 µg/mL of AgNPs-cinnamon. Figure S7 Temporal progress of the AgNPs formation evaluated by the absorbance at plasmonic band wavelength. Figure S8 Plasmonic band of AgNPs-cinnamon as a function of silver nitrate concentration.
