# Supplementary material for: Synergistic Antibacterial Properties of Silver Nanoparticles and Its Reducing Agent from Cinnamon Bark Extract
Source: Bioengineering (Basel). 2024 May 20;11(5):517. doi: 10.3390/bioengineering11050517 (PMC11117492; doi:10.3390/bioengineering11050517)
Supplement: Supplementary file 1 [file bioengineering-11-00517-s001.zip › bioengineering-2975783-supplementary.pdf]

Supplementary Material: Synergistic antibacterial properties of silver nanoparticles and its reducing agent from cinnamon bark extract

Araceli Granja Alvear [1], Nayely Pineda-Aguilar [2], Patricia Lozano [3], Cristóbal Lárez-Velázquez [4], Gotfried Suppan[1], Salomé Galeas [5], Alexis Debut [6], Karla Vizuete[6], Lola De Lima [1], Juan Pablo Saucedo-Vázquez [1], Frank Alexis [7,*], Floralba López [1, *].

[1] CATS Research Group, School of Chemical Sciences Engineering, Yachay Tech University, Urcuquí, 100119, Ecuador; flopez@yachaytech.edu.ec

[2] Centro de Investigación de Materiales Avanzados CIMAV-Monterrey, Monterrey, 64630, México

[3] Centro de Investigaciones en Ciencias Microbiológicas. Instituto de Ciencias. Universidad Autónoma de Puebla. Puebla, 72570 México

[4] Laboratorio de Polímeros, Departamento de Química, Facultad de Ciencias, Universidad de Los Andes, Mérida, 5101, Venezuela.

[5] Laboratorio de Nuevos Materiales (LANUM), Escuela Politécnica Nacional, Quito, 170143, Ecuador.

[6] Centro de Nanociencia y Nanotecnología, Universidad de las Fuerzas Armadas ESPE, Sangolqui, 171523, Ecuador

[7] Departamento de Ingeniería Química, Colegio de Ciencias e Ingeniería, Instituto de Energía y Materiales, Instituto de Microbiología, Universidad San Francisco de Quito (USFQ), Quito, 170901, Ecuador; falexis@usfq.edu.ec

A qualitative screening for secondary metabolites in cinnamon extract was conducted through various colorimetric tests. The emulsion obtained (Figure S1) from the steam distillation was analyzed by several methods to identify flavonoids, phenolic compounds, tannins, coumarins, alkaloid, and protein. The description of the developed methods and the results of such analyzes are presented below.

S1. Methodology
Characterization of cinnamaldehyde aqueous extract

- UV-Vis spectra was determined using the cinnamaldehyde aqueous extract using an Analityk Jenna Spectracord S200 diode array spectrometer in the range of 190-700 nm.

- HPLC was recorded in a HPLC Thermo UltiMate 3000 equipped with a diode-array detector (DAD) and a C-18 column for HPLC Hypersil GOLD™ (150 mm× 4.6 mm, 5 µ particle size). The mobile phase was comprised of an acetonitrile:water 60:40 mixture at a flow rate of 1 mL/min at room temperature. The injection volume was 5 µL, and the detection wavelength was set at 210 nm. The retention time was used for qualitative analysis.
- To determine secondary metabolites in the cinnamon extract, a qualitative screening was carried out using classical colorimetric tests.

Determination of Flavonoids

- Concentrated H_2SO_4 Test: To confirm the presence of flavonoids in the cinnamon extract, 0.5 mL of concentrated sulfuric acid was added to 1 mL of the extract.
- Shinoda's Test: 5 mL of cinnamon extract with a combination of magnesium ribbon fragments and 0.5 mL of concentrated hydrochloric acid. The objective was to observe a distinctive color shift from pink to crimson, indicative of the presence of flavanol glycosides.

Detection of simple Phenolic Compounds

- Iodide test: The iodide test involved adding three drops of diluted iodine solution to 1 mL of cinnamon extract. The presence of phenols was expected to be validated by a change to a red color in the solution.
- The Ferric Chloride Test: involved the addition of four drops of a 5% ferric chloride solution to 1 mL of cinnamon extract. The confirmation of the presence of phenols was contingent upon a color change to either bluish-black or dark green.
    - *Detection of Tannins* 10% NaOH test: In 1 mL of the extract added of 4 mL of sodium hydroxide solution, followed by thorough shaking. The confirmation of hydrolysable tannins was expected through the formation of an emulsion.
- Braymer's Test: 1 mL of the cinnamon extract underwent a procedure involving the addition of 3 mL of distilled water and three drops of a 10% ferric chloride solution. The confirmation of tannins was expected through a color change to green or blue.

Detection of Coumarins

- NaOH Test: In 1 mL of extract were added 0.5 mL of 10% sodium hydroxide solution and 0.5 mL of chloroform. If the solutions turn yellow, the presence of coumarins will be confirmed.

Detection of Alkaloids

- Dragendroff's Test: 1 mL of cinnamon bark extract underwent the addition of 1 mL of Dragendroff's reagent. The presence of alkaloids was confirmed through the formation of a reddish-brown solution.

- Wagner's Test: 1 mL of the extract underwent the addition of two drops of Wagner's reagent, which were then placed along the sides of a test tube. The confirmation of alkaloids was expected through the formation of a precipitate with a reddish or brown color.
- The Lugol's Test, designed to detect the presence of phenolic compounds by inducing a brown color change in the extract, was conducted using 1 mL of the sample and four drops of Lugol reagent.

Determination of Proteins

- Biuret Test: 1 mL of cinnamon extract underwent the addition of two drops of biuret reagent. The Biuret Test is done to show the presence of peptide bonds of the proteins. These bonds will make the blue Biuret reagent turn purple if positive.

S2. Results

Characterization of cinnamaldehyde aqueous extract

- UV-Vis spectrum of the cinnamon bark extract shows the spectral characteristics of trans-cinnamaldehyde with a maximum of absorption at 292 nm (Figure S1).

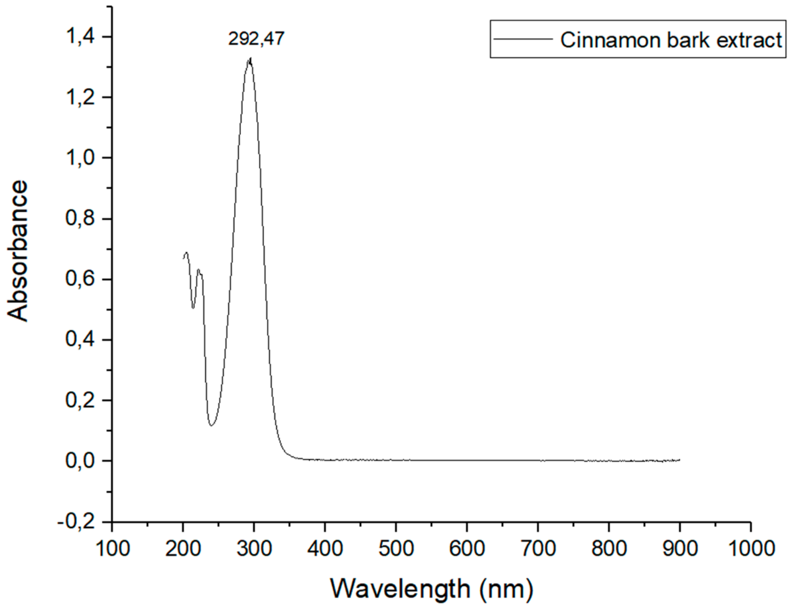

Figure S1. UV-Vis spectrum of cinnamon bark extract.

- The results of the HPLC analysis showed that we have a major component in almost 100% of concentration, such peak is assigned to cinnamaldehyde since it was compared with the retention time of a cinnamaldehyde standard (Figure S2).

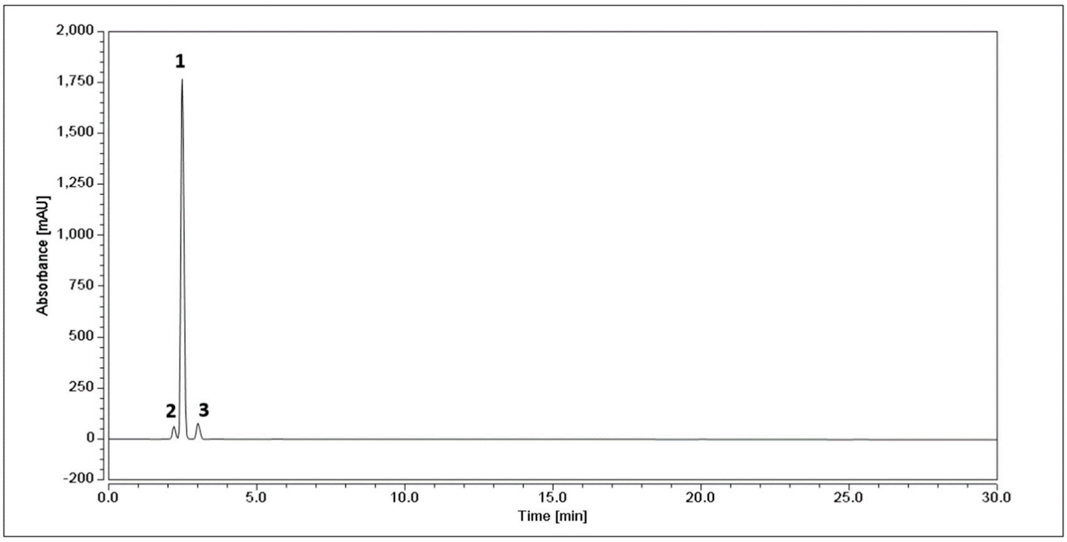

Figure S2. Chromatogram of cinnamon bark extract on the HILIC column. Peak 1 represents cinnamaldehyde and peaks 2 and 3 represent two minor components of the extract.

The results of the determination of secondary metabolites are shown in Table S1 and a conclusion of the entire screening is presented after the table.

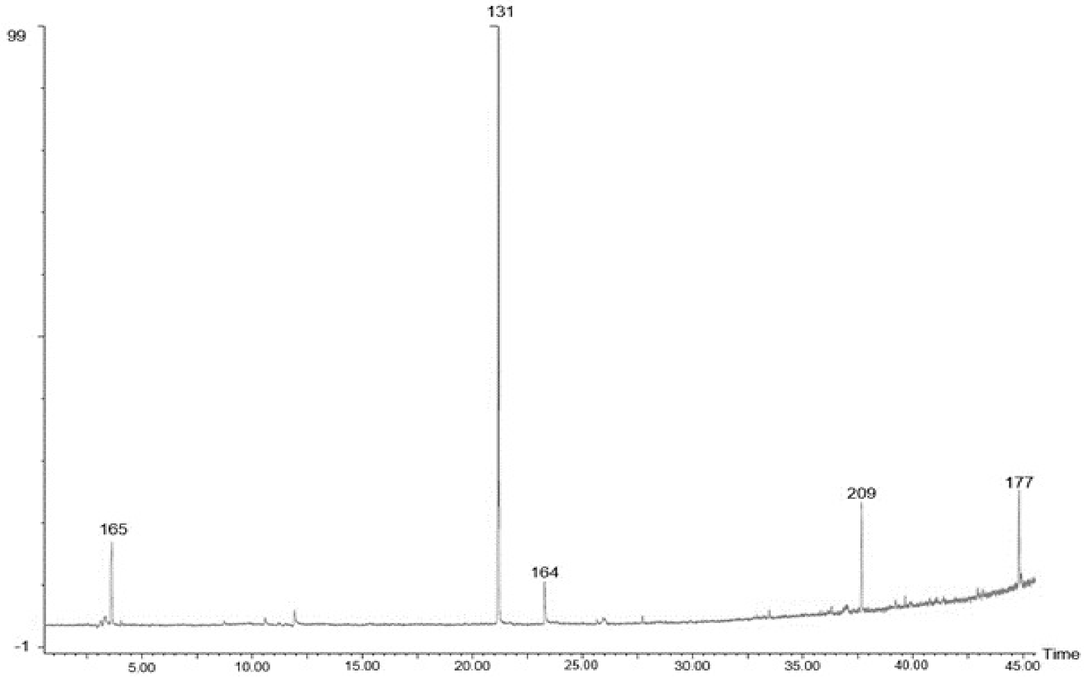

Figure S3. GC chromatogram showing the characteristic base peak of cinnamaldehyde m/z = 131 at a retention time of 22.3 min.

Table S1. Results of the determination of secondary metabolites in the cinnamon bark. extract.

Secondary Metabolite	Test	Result	Figure	Reference
NA	Cinnamon bark extract		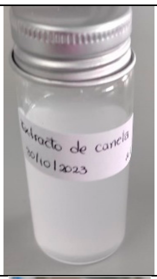	
Flavonoids	Concentrated H_2SO_4 Test	Positive as the solution turned orange-red.	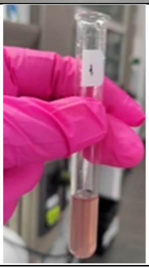	SR1
	Shinoda's Test	Negative	--	SR2
Phenols	Iodide test	Negative	--	SR3
	Ferric Chloride Test	Negative	--	SR4
Tanins	10% NaOH test	Negative	--	SR3
	Braymer's Test	Negative	--	SR5
Coumarins	NaOH Test	Negative	--	SR3
Alkaloids	Dragendroff's Test	Positive as evident by the change of color.	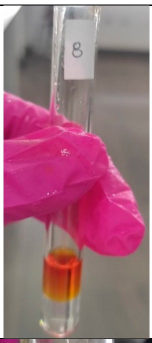	SR6
	Wagner's Test	Positive result, as evidenced by the formation of precipitate.	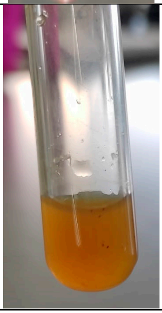	SR3
Carbohydrates	Lugol's Test	Negative	--	SR3

Proteins	Biuret Test	Negative	--	SR6
Ammonium		Positive	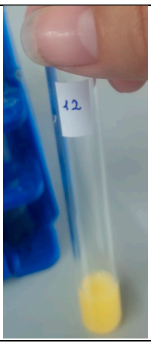	SR7

Conclusions on the determination of secondary metabolites

A qualitative screening for secondary metabolites in cinnamon extract was conducted through various colorimetric tests (Figure S4). The presence of flavonoids was confirmed by the positive outcome of the concentrated sulfuric acid test, which resulted in an orange-red solution. However, the Shinoda's Test yielded a negative result, as there was no observable pink to crimson color change. Simple phenolic compounds were not detected, as evidenced by the negative outcomes in iodide test, Lugol's test and the ferric chloride test, with no transient red or bluish black/dark green color changes, respectively. Tannins, coumarins, and proteins also showed negative results in their respective tests. Alkaloids were positively identified through the Dragendroff's test and Wagner's test, where reddish-brown precipitates formed. Lastly, the presence of ammonium was confirmed by the positive outcome of the Nessler test, with the solution turning yellow. It's important to note that

these tests provide qualitative insights, and a more in-depth quantitative analysis is necessary for accurate concentration assessments of these secondary metabolites.

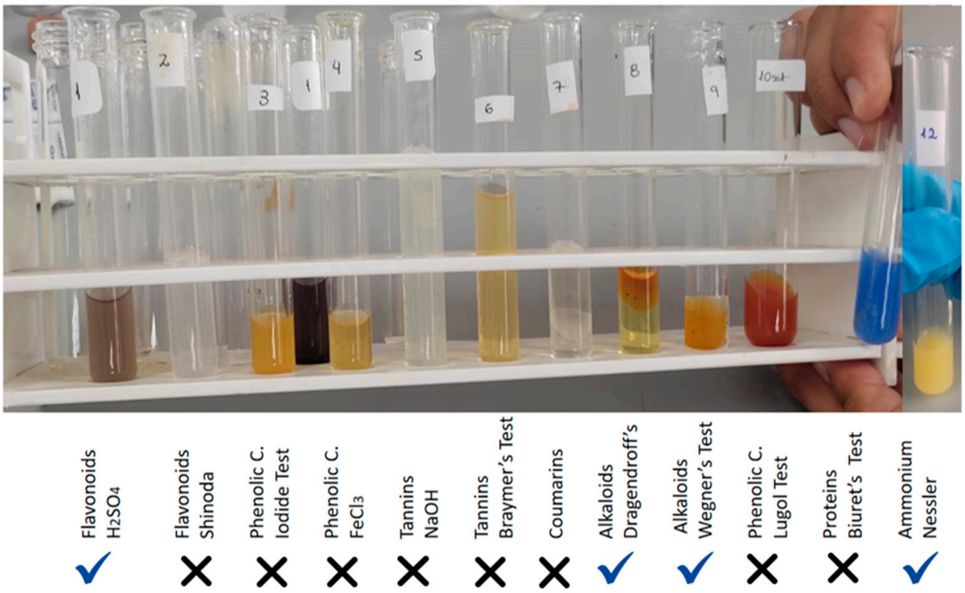

Figure S4 Phytochemical screening of qualitative tests to secondary metabolites.

Antibacterial Assays

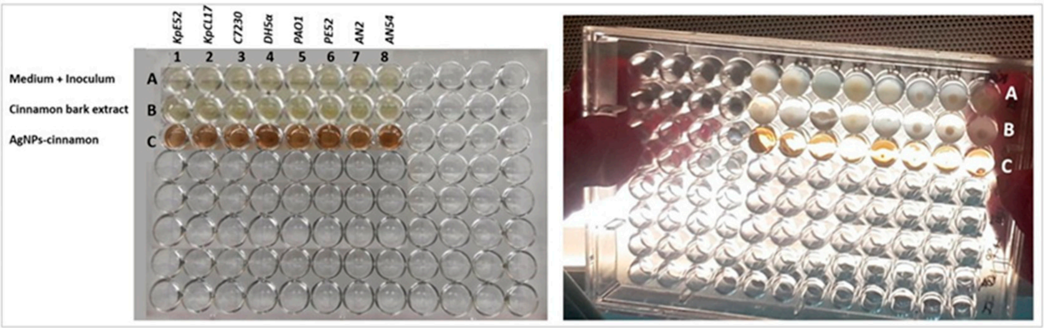

Figure S5 ELISA microplate of broth microdilution method to analyze the antimicrobial activity of silver nanoparticles for the tested bacteria.

In Figure S5 the ELISA microplate corresponds to the broth microdilution method, in which the bacterial growth is presented. In this figure the effect of presence of AgNPs-cinnamon against the evaluated bacteria can be observed.

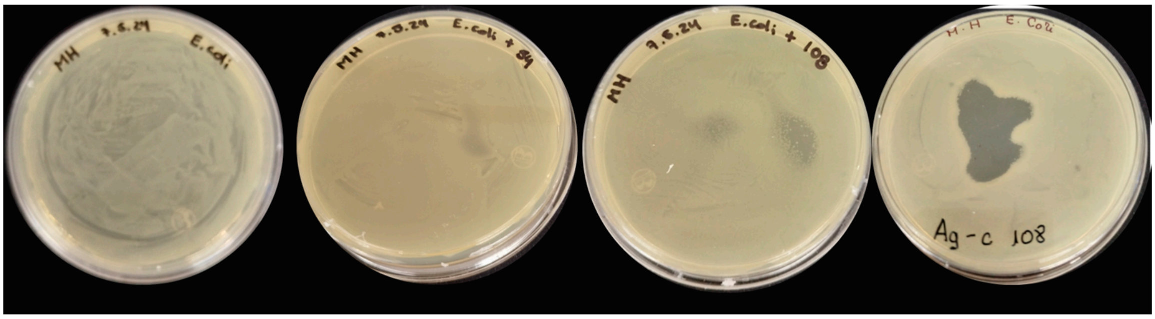

Figure S6 Bacterial viability at 54, 108, and 540 μg/mL of AgNPs-cinnamon.

The effect of the presence de AgNPs-cinnamon on bacterial viability determined by mixing the AgNPs-cinnamon and E. coli bacteria is shown in Figure S6. The mixtures were plated, and the viability as a function of the AgNPs-cinnamon concentration was determined. The inhibition region observed demonstrates the effectivity of the AgNPs-cinnamon.

The plots depicted in Figure S7 show the temporal progress of the formation of AgNPs-cinnamon. As expected, the continuous increase of the plasmonic band can be attributed to the increase in the AgNPs-cinnamon concentration. On the other hand, the AgNPs-cinnamon concentration effect on the intensity of the plasmonic band is shown in Figure S8.

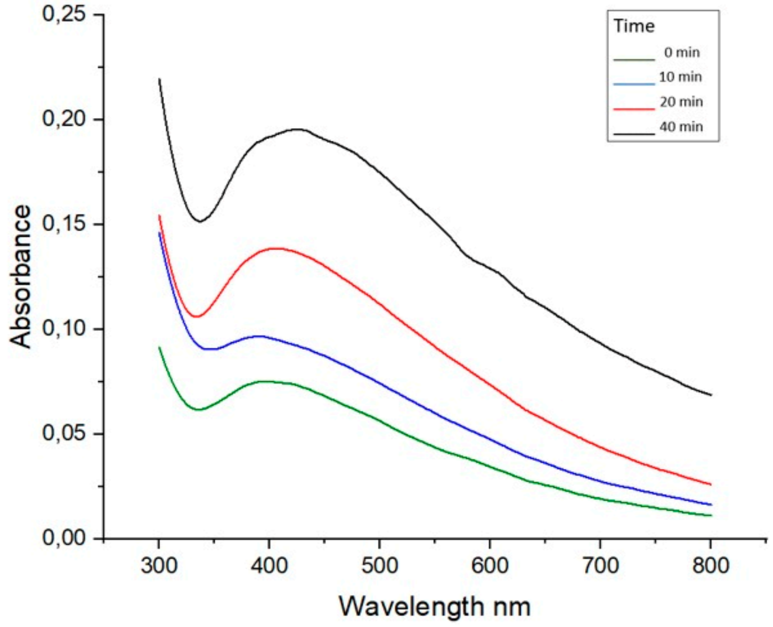

Figure S7 Temporal progress of the AgNPs formation evaluated by the absorbance at plasmonic band wavelength.

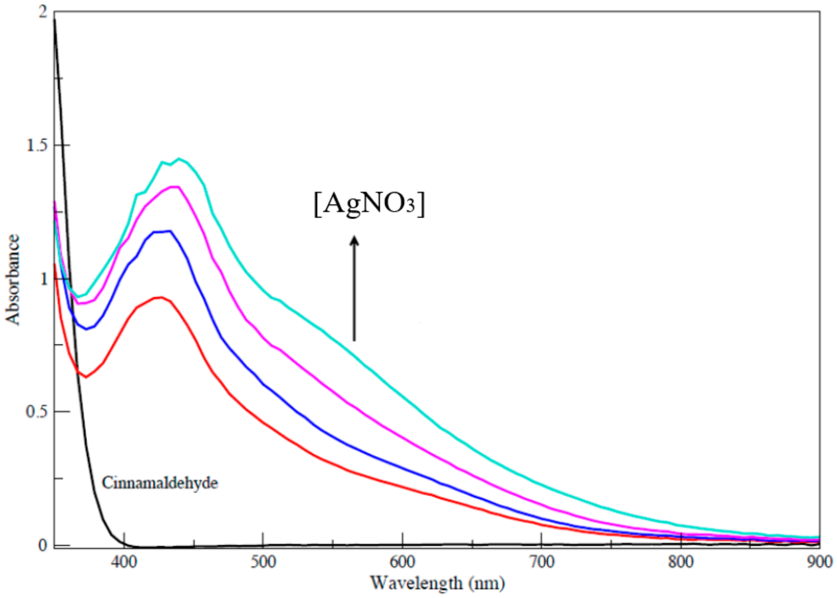

Figure S8 Plasmonic band of AgNPs-cinnamon as a function of silver nitrate concentration.

S3. References

- **SR1**. Tyagi T. Phytochemical Screening of Active Metabolites Present in Eichhornia Crassipes (Mart.) Solms and Pistia stratiotes (L.): Role in Ethanomedicine. Asian Journal of Pharmaceutical Education and Research. 2017; 6(4):40-56.
- **SR2**. Raaman N. Phytochemical Techniques. New India Publishing Agency, New Delhi, 2006, 19-24.
- **SR3**. Singh V, Kumar R. Study of Phytochemical Analysis and Antioxidant Activity of Allium sativum of Bundelkhand Region. International Journal of Life Sciences Scientific Research. 2017; 3(6):1451-1458.
- **SR4**. Tiwari P, Kumar B, Kaur M, Kaur G, Kaur H. Phytochemical screening and Extraction: A Review. Internationale Pharmaceutica Sciencia. 2011; 1(1):98-106.
- **SR5**. Uma KS, Parthiban P, Kalpana S. Pharmacognostical and Preliminary Phytochemical Screening of Aavaarai Vidhai Chooranam. Asian Journal of Pharmaceutical and Clinical Research. 2017; 10(10):111-116.
- **SR6**. Silva GO, Abeysundara AT, Aponso MM. Extraction methods, qualitative and quantitative techniques for screening of phytochemicals from plants. American Journal of Essential Oils and Natural Products. 2017; 5(2):29-32.
- **SR7**. Ammonia determination in bottled water using spectrophotometer: comparison between Nessler and Berthelot methods. Sasongko, Ashadi JST (Jurnal Sains dan Teknologi) 7.1 (2018): 126-134.
